# Stenocarpin

**Published:** 1887-10

**Authors:** Wm. H. Mitchell

**Affiliations:** Bergen Point, N. J.


### STENOCARPIN.

BY WM. H. MITCHELL, D. D. S., BERGEN POINT, N. J.

This drug, but recently introduced into the materia medica of dentistry, was first brought to the attention of the writer by Allan W. Seward, M. D., of Bergen Point, N. J., about the first of April of this year.

It was discovered by Dr. Seward in making an analysis of the leaves of the tree known in Louisiana as the Tear Blanket Tree, and had been brought to his attention by M. Goodman, V. S., of Bayou Sara, of that State, who had noticed that a poultice of the leaves, which he had applied to the fetlock of a horse that had been injured, possessed anæsthetic properties. He sent a quantity of the leaves to Dr. Seward, who discovered the alkaloid, which he has christened Stenocarpin, and which has for its formula $C_{20}H_{21}NO_3$.

The Tear Blanket Tree grows along the banks of the rivers and streams of the lower Mississippi Valley, and attains a height of from thirty-five to forty feet, the branches spreading from thirty to thirty-five feet. The bark is smooth, and is covered with bunches of sharp spines that spring from a parent thorn, the bunch being quite flexible, but exceedingly difficult to detach from the tree, from which it grows at a right angle. These thorns often reach six inches in length. The tree bears a bean which grows in pods from eight to ten inches in length.

Dr. Seward mentions that in his experiments he had found it much superior to cocaine, and suggested that it might be a useful addition to the list of dental medicants.

Since April I have used it as an obtundent of sensitive dentine, and have had remarkable success with it. I made mention of it at the First District Dental Society of New York, at the June meeting, and since that time quite a number of practitioners of dentistry have been using the drug with marked success, wherever its anæsthetic properties were sought in obtunding sensitive dentine, soothing exposed pulps, or removing pulps and small growths from the surface of the mucous membrane of the mouth.

Dr. J. Herbert Claiborne, Jr., of New York, has witnessed wonderful results from its use in Ophthalmic Surgery, and reports the removal, from the forehead of a patient, of a sebaceous tumor of oval form, about one and one-fourth by three-fourths of an inch in size. This was done by saturating absorbent cotton with the two per cent. solution, which was placed over and around the growth. After a period of twenty minutes the sensation of the part had disappeared, and at the end of half an hour the operation was commenced and finished, removing the growth entire without any pain, save at one time when, in the deepest part of the operation, a slight sensation was perceived. A quantity of the solution was immediately applied, and the operation completed. A suture was passed through the wound, and it healed by first intention.

Those who have used the drug state they are highly pleased with its effects, and that there is a long future for it. It is a direct antagonist of morphine and opium, ten drops of the two per cent. solution neutralizing one grain of morphine or six of opium. Hypodermically administered, it is especially useful as an aid in extraction of teeth, when it is not desirable to administer a general anæsthetic.

